# Implication and challenges of mobile health and blockchain technology for remote patient monitoring

**DOI:** 10.1016/j.jtumed.2023.05.018

**Published:** 2023-06-07

**Authors:** Manik Sharma

**Affiliations:** Department of CSA, DAV University Jalandhar, India

In recent years, several remote monitoring frameworks have been designed by various researchers using artificial intelligence, transfer learning, cloud computing, fog computing, mobile computing, soft computing, and drone technology.^1^, ^2^, ^3^ Mobile devices are required to complete the remote monitoring framework. Amidst several preventative steps, the security and privacy of the data have remained a challenge for these smart healthcare models. The use of blockchain technology has soared in recent years and has offered the required solution for the same. However, one of the key questions to consider is whether the use of blockchain can alleviate all the security concerns connected with Remote Patient Monitoring (RPM). The initial objective of this work is to provide a quick overview of mhealth and blockchain technologies, as well as their relevance in the healthcare industry. Finally, the ramifications, as well as the benefits and risks, of incorporating mhealth and blockchain in designing the RPM system have also been addressed.

## Remote patient monitoring

It is an innovative supervision approach in which a physician sitting far away from the patient can remotely monitor the patient's condition. In the last few years, several RPM devices have been designed to observe, report, and analyze a patient's condition (acute or chronic diseases) from outside of health clinics or medical centres. When utilized properly, RPM devices boost in-person attention, allowing healthcare providers to fully comprehend the patient's symptoms, rather than during the in-person visit. The use of these devices helps healthcare professionals in making make proactive clinical decisions. Furthermore, these devices assist victims (patients) in engaging with these devices so that they can record and check their health status regularly.

## Mobile health and blockchain

The m-health services are proven to benefit self-diagnosis, treatment, remote monitoring, telemedicine, and chronic illness. The m-health services are part of mobile computing technology in which a user can self-diagnose him or herself using different kinds of mobile apps. The users of m-health services can use their smartphones or tablet to capture and analyze their health data without taking a clinician's help. The users may utilize wearable gadgets and other mobile technology to track and manage their health status daily without having to contact their doctors. In medical sciences, the patient's data is of utmost importance and as per government policies, it needs to be fully secured. However, while using m-health services, the data is being regularly transmitted between the patient and the third party (owner of mobile apps), which seems to be a serious concern in the implementation of mhealth services.

Blockchain is a type of advanced data structure that aids in the creation of distributed databases in which data is organized into groups or blocks. The blocks are interlinked with one another. The chain of these data blocks has been named blockchain. As the data stored in these blocks have been shared across all nodes on the network, therefore, everyone (each peer) connected to the network has access to the data stored in the blockchain.^4^ Moreover, based on participation, the blockchain can be categorized as public, private, or consortium. In a public blockchain (Bitcoin, Ethereum), anyone can join, whereas a proper invitation is required to join a private and consortium blockchain. The immutability of transactions, cryptography, smart contracts, and distributed nature of this technology make it more resilient and beneficial in a variety of sectors, including finance, healthcare, data management, real estate, voting, non-fungible tokens, and government policies. The scientific literature revealed that as far as temporal factors are considered, most blockchain-related work has only concentrated on two key parameters (data management and interoperability). Similarly, spatial research uncovered that the United States and China have investigated the majority of blockchain-related work in the healthcare sector.

## Implications of mobile computing and blockchain in healthcare

Over the next ten years (2021–2030), the current value of the mhealth industry is anticipated to rise at a 30 percent compound annual growth rate. It has been perceived that in the coming years, the key services (remote monitoring and patient tracking services) of mobile apps will surely assist in accomplishing lucrative growth.^5^ The health-related data (temperature, blood pressure, saturation rate, heart rate, etc.) captured through m-health apps can be examined to reveal the physical and mental state of the victims.

The use of blockchain (a new decentralized mechanism) can effectively mitigate the risk of data hazards. It seems to be a robust asset in competing for security and efficiency challenges in designing smart and remote healthcare systems.^5^ Moreover, the use of blockchain can also help in uploading and maintaining a single record (interstate medical licensure) of healthcare professionals so that their authentication and certification can be easily accessed and approved. The complete record of a patient including his blood pressure, sugar level, heart rate, mental state and even more complicated data of CT scan, MRI images, and other physiological signals can be stored as a single record in the blockchain. This information will help healthcare professionals remotely monitor the precise state of the patients. Moreover, based on the circumstances, a physician may also request other experts to scan the contemporary state of the individuals using the stored information. The traceability feature of the blockchain helps healthcare professionals to examine the kind of drugs and treatments recommended to the patient in the past. The existing research witnessed that this decentralized technology has been effectively used to solve distinct real-life healthcare-related applications viz. medical data management, blood donation, clinical trial, health insurance, drug-supply-chain, patient tracking, etc.^6^^,^^7^

## Disaster management and outbreaks

The need for remote patient monitoring and real-time information has been explicitly highlighted by the current pandemic (COVID-19). The blockchain-based m-health services have the potential to reinforce communication and health management services for remote patients as well as for the victims of disasters and outbreaks. These services offer paperless and rapid action to deal with disaster management.^8^ The blockchain-based m-health services can be utilized to offer digital intervention to ameliorate the physical and psychological impact of the disaster which would subsequently hold back the triggering effect of the disastrous conditions and helps in reducing the number and intensity of infections.^9^ These kinds of mHealth services can also be utilized to educate and train the victims to make them healthy and safe during disasters and pandemics.

## Potential consequences of blockchain in remote monitoring

In remote monitoring, the data has to be regularly transmitted between the patient and the healthcare professionals. However, healthcare data was found to be a more lucrative target for hackers. However, as per government regulation, during the transmission, the patient's data need to be completely secured and protected. The use of blockchain technology can assist in recording (in the form of an unalterable log) all the transactions that have been performed between the patients and the healthcare professionals during the remote monitoring process.^10^ Moreover, the use of blockchain also provides the facility of smart contracts (segments of code) which are automatically triggered based on some predefined circumstance and events. With blockchain technology, the precautions and the prescribed treatment for remote patients can also be maintained in the form of metadata so that it can be adequately audited in the future if required. However, due to the unstructured nature of data, data standardization may be of concern in this complete scenario. Despite, major security concerns, some researchers have pointed out that to some extent, the blockchain implementation is still vulnerable to DNS and other cyber-attacks. In addition, the scalability of the blockchain also seems to be the bone of contention. At last, due to the large data volume, one may also face some issues of slow processing and high energy consumption.^11^

To summarize, despite its widespread application, various factors such as social acceptability, regulatory frameworks, and interoperability continue to impede the optimal implementation of blockchain technology. To alleviate these issues, there is a need to hybrid blockchain technology with mobile computing, the Social Internet of Things (SIoT), and fog computing. The hybridization of blockchain, SIoT, and fog computing will aid in improving the slow transaction processing times and rate of adoption. The integration of blockchain and machine learning together will facilitate the precise diagnosis of remote patients. Moreover, natural language processing (key application of machine learning) can overcome the issue of language or cultural differences present in digital records of blockchain. However, still, to maintain the high pace of data encryption, a lot of computational power will be used, which comes with a significant environmental cost. Finally, the cost of implementation will be still a matter of concern.

## Source of funding

This research did not receive any specific grant from funding agencies in the public, commercial, or not for-profit sectors.

## Conflict of interest

The authors have no conflict of interest to declare.

## Ethical approval

This is purely a technical article based on mobile computing and blockchain.
